# Antibiofilm Activity of *Actinobacillus pleuropneumoniae* Serotype 5 Capsular Polysaccharide

**DOI:** 10.1371/journal.pone.0063844

**Published:** 2013-05-14

**Authors:** Michael T. Karwacki, Daniel E. Kadouri, Meriem Bendaoud, Era A. Izano, Vandana Sampathkumar, Thomas J. Inzana, Jeffrey B. Kaplan

**Affiliations:** 1 Department of Oral Biology, New Jersey Dental School, Newark, New Jersey, United States of America; 2 Department of Biomedical Sciences and Pathobiology, Virginia-Maryland Regional College of Veterinary Medicine, Virginia Tech, Blacksburg, Virginia, United States of America; 3 Department of Biology, American University, Washington, District of Columbia, United States of America; Université d’Auvergne Clermont 1, France

## Abstract

Cell-free extracts isolated from colony biofilms of *Actinobacillus pleuropneumoniae* serotype 5 were found to inhibit biofilm formation by *Staphylococcus aureus*, *S. epidermidis* and *Aggregatibacter actinomycetemcomitans*, but not by *A. pleuropneumoniae* serotype 5 itself, in a 96-well microtiter plate assay. Physical and chemical analyses indicated that the antibiofilm activity in the extract was due to high-molecular-weight polysaccharide. Extracts isolated from a mutant strain deficient in the production of serotype 5 capsular polysaccharide did not exhibit antibiofilm activity. A plasmid harboring the serotype 5 capsule genes restored the antibiofilm activity in the mutant extract. Purified serotype 5 capsular polysaccharide also exhibited antibiofilm activity against *S. aureus*. *A. pleuropneumoniae* wild-type extracts did not inhibit *S. aureus* growth, but did inhibit *S. aureus* intercellular adhesion and binding of *S. aureus* cells to stainless steel surfaces. Furthermore, polystyrene surfaces coated with *A. pleuropneumoniae* wild-type extracts, but not with capsule-mutant extracts, resisted *S. aureus* biofilm formation. Our findings suggest that the *A. pleuropneumoniae* serotype 5 capsule inhibits cell-to-cell and cell-to-surface interactions of other bacteria. *A. pleuropneumoniae* serotype 5 capsular polysaccharide is one of a growing number of bacterial polysaccharides that exhibit broad-spectrum, nonbiocidal antibiofilm activity. Future studies on these antibiofilm polysaccharides may uncover novel functions for bacterial polysaccharides in nature, and may lead to the development of new classes of antibiofilm agents for industrial and clinical applications.

## Introduction

Biofilm is the predominant mode of growth for bacteria in most natural, industrial and clinical environments [1]. Biofilms typically contain millions of tightly-packed cells encased in a polymeric matrix attached to a tissue or surface. The biofilm mode of growth protects bacteria from cell stressors such as desiccation, predators and antibiotics. Biofilms cause corrosion and biofouling of industrial equipment and chronic infections in clinical settings [2]. New methods for treating and preventing biofilm formation are being sought.

Many biofilm bacteria secrete molecules such as quorum-sensing signals [3], [4], surfactants [5], enzymes [6], [7], polysaccharides [8], and D-amino acids [9] that function to regulate biofilm architecture or mediate the release of cells from biofilms during the dispersal stage of the biofilm life-cycle. These compounds often exhibit broad spectrum biofilm-inhibiting or biofilm-dispersing activity when tested against biofilms cultured in vitro. Such compounds may represent a novel source of antibiofilm compounds for technological development [10].

Previous studies showed that colony biofilms may be a useful source of novel antibiofilm compounds [11]. Colony biofilms are lawns of bacterial cells cultured directly on an agar surface or on a semipermeable membrane that sits on an agar plate [12]. Colony biofilms have been shown to exhibit many properties characteristic of broth-cultured biofilms, including high cell density, extracellular matrix production, spatially dependent microbial growth, chemical gradients, and reduced susceptibility to antibiotics [13]-[18]. Cell-free extracts isolated from colony biofilms [11], or from biofilms cultured in a continuous-flow fermentor that produces a similarly high amount of biofilm biomass [19], have been shown to be enriched for soluble molecules produced within the biofilm matrix.

In the present study we screened a panel of 12 colony biofilm extracts isolated from various bacteria for their ability to inhibit *Staphylococcus aureus* biofilm formation in a 96-well microtiter plate assay. We found that colony biofilm extracts isolated from *Actinobacillus pleuropneumoniae* serotype 5 inhibited *S. aureus* biofilm formation without inhibiting *S. aureus* growth. Here we present results suggesting that the nonbiocidal antibiofilm activity in the *A. pleuropneumoniae* extract is due to high-molecular-weight serotype 5 capsular polysaccharide.

## Methods

### Bacterial Strains, Media and Growth Conditions

The bacterial strains used in this study are listed in Table 1. For solid media, Tryptic Soy agar was used for *A. pleuropneumoniae*, *A. actinomycetemcomitans* and *H. influenzae*, sheep blood agar (Catalog No. 221239; Becton, Dickinson and Co.) was used for staphylococci, and LB agar was used for all other bacteria. For broth cultures, Tryptic Soy broth supplemented with 6 g/L yeast extract and 8 g/L glucose was used for *A. pleuropneumoniae*, *A. actinomycetemcomitans*, *H. influenzae* and staphylococci, and LB broth was used for all others. *A. pleuropneumoniae* cultures were supplemented with 10 mg/L NAD. *H. influenzae* cultures were supplemented with 10 mg/L NAD and 10 mg/L hemin. Plasmid-harboring *A. pleuropneumoniae* strains were cultured in 80 mg/L spectinomycin. *A. pleuropneumoniae* and *A. actinomycetemcomitans* cultures were incubated in 10% CO_2_, whereas all other cultures were incubated in air. *P. carotovorum* cultures were incubated at 28°C, while all others were incubated at 37°C.

**Table 1:** Bacterial strains.

Species	Strain	Source or reference*
*Acinetobacter lwoffii*	ATCC 15309	ATCC
*Acinetobacter haemolyticus*	ATCC 19002	ATCC
*Actinobacillus pleuropneumoniae*	J45 (wild-type; serotype 5a)	[21]
*Actinobacillus pleuropneumoniae*	J45-100 (J45 Δ*cps5ABC*; capsule-mutant)	[29]
*Actinobacillus pleuropneumoniae*	J45-100+ pJMLCPS5 (genetically-complemented capsule-mutant)	[29]
*Aggregatibacter actinomycetemcomitans*	CU1000	[36]
*Citrobacter freundii*	ATCC 43864	ATCC
*Enterobacter aerogenes*	ATCC 35029	ATCC
*Enterobacter amnigenus*	ATCC 51816	ATCC
*Haemophilus influenzae*	NJ9725	[37]
*Klebsiella pneumoniae*	ATCC BAA-1705	ATCC
*Lactococcus lactis*	525A	PIC
*Pectobacterium carotovorum*	ATCC 15713	ATCC
*Proteus vulgaris*	ATCC 8427	ATCC
*Pseudomonas aeruginosa*	PP	Environmental isolate
*Staphylococcus aureus*	SH1000	[38]
*Staphylococcus epidermidis*	NJ9709	[35]

* ATCC, American Type Culture Collection, Manassas VA, USA; PIC, Presque Isle Cultures, Erie PA, USA.

### Preparation of Colony Biofilm Extracts

A 100-µl aliquot of an overnight broth culture (>10^8^ CFU) was spread onto the surface of a 100-mm-diam agar plate using a sterile glass spreader. The plate was incubated for at least 48 h until a robust lawn of microbial growth (colony biofilm) developed. Thereafter, the cell paste was scraped from the surface of the plate using a plastic inoculating loop or plastic cell scraper, and the cells were transferred to a microcentrifuge tube containing 750 µl of saline (0.9% NaCl). The tubes were mixed by vortex agitation for 10 min, and the cells were pelleted by centrifugation. The supernatant was sterilized by passage through a 0.22-µm pore-size filter, and the resulting colony biofilm extract was stored at 4°C until use.

### Screening Colony Biofilm Extracts for Antibiofilm Activity

*S. aureus* inocula were prepared from 18-h-old agar colonies as previously described [20]. A volume of 180 µl of inoculum (ca. 10^4^–10^5^ CFU/ml) was transferred to the well of a tissue-culture-treated polystyrene microtiter plate (Falcon no. 353047). A total of 20 µl of colony biofilm extract, or 20 µl of saline as a control, was mixed with the inoculum and the plate was incubated statically at 37°C. After 18 h, biofilms were rinsed with water and stained for 1 min with 200 µl of Gram’s crystal violet. Wells were then rinsed with water and dried. The amount of crystal violet binding was quantitated by destaining the wells for 10 min with 200 µl of 33% acetic acid, and then measuring the absorbance of the crystal violet solution in a microplate spectrophotometer set at 595 nm.

### Physical and Chemical Analyses of *A. pleuropneumoniae* Colony Biofilm Extract

Size-exclusion filtration was carried out using a Microcon centrifugal concentrator (Millipore) with a 100-kDa molecular weight cut-off filter. For enzymatic treatments, extracts were incubated for 1 h at 37°C with 100 mg/L DNase I, RNase A, porcine pancreatic lipase or proteinase K (Sigma-Aldrich) or 10 mg/L dispersin B (Kane Biotech). Controls consisted of mock-treated extracts, or enzymes alone with no extract. For sodium metaperiodate treatment, 0.1 vol of 100 mM sodium metaperiodate was added to the extract, and the extract was incubated at 37°C for 1 h. Controls consisted of mock-treated extract and sodium metaperiodate alone with no extract. Following all treatments, extracts and controls were incubated at 100°C for 10 min prior to testing in the *S. aureus* biofilm assay described above.

### Purification of *A. pleuropneumoniae* Serotype 5 Capsular Polysaccharide

Capsular polysaccharide was purified from broth cultures of *A. pleuropneumoniae* strain J45 by Cetavlon precipitation of culture supernatant, extraction of the precipitate with NaCl and aqueous phenol, and Sepharose CL-4B gel filtration chromatography as previously described [21].

### Cell Binding Assay

A single-cell suspension of *S. aureus* (ca. 10^6^–10^7^ CFU/ml) was prepared in fresh broth using a filtration protocol as previously described [22]. The cell suspension was supplemented with 10% *A. pleuropneumoniae* colony biofilm extract, or 10% saline as a control, and then aliquots of cells (0.5-ml each) were transferred to 1.5-ml polypropylene microcentrifuge tubes. Stainless steel rods (0.6-mm diam×13-mm length) were placed in the tubes, and the tubes were incubated at 37°C. After 30 or 60 min, rods were removed from the tubes, rinsed three times with saline, and transferred to 15-ml conical centrifuge tubes containing 1 ml of saline. The rods were sonicated on ice (2×30 sec) using an IKA Labortechnik sonicator set to 50% power and 50% duty cycle. CFUs in the sonicate were quantitated by dilution plating.

### Intercellular Adhesion Assay

*S. aureus* was cultured in 17-mm ×100-mm glass tubes in 2 ml of broth. The broth was supplemented with 7% (by vol) *A. pleuropneumoniae* colony biofilm extract isolated from strain J45 or J45-100, or with 7% saline as a control. Bacteria were incubated with shaking (200 rpm). After 7 h, tubes were incubated statically for 10 min and then photographed.

### Surface Coating Assay

A volume of 25 µl of *A. pleuropneumoniae* colony biofilm extract, or 25 µl of saline as a control, was transferred to the center of a well of a 24-well tissue-culture-treated polystyrene microtiter plate (Falcon no. 353047). The plate was incubated at 37°C for 30 min to allow complete evaporation of the liquid. The wells were then filled with 1 ml of broth containing 10^4^ to 10^5^ CFU/ml of *S. aureus*. After 18 h, wells were rinsed with water and stained with 1 ml of Gram’s crystal violet. Stained biofilms were rinsed with water and dried, and the wells were photographed.

### Microscopy

*A. pleuropneumoniae* inocula were prepared from 24-h-old agar colonies as previously described [20]. Bacterial inocula were diluted in fresh broth to 10^4^–10^5^ CFU/ml. Aliquots of diluted cells (250 µL each) were pipetted onto the surface of sterile glass slides, and the slides were placed inside a Petri dish. After incubation for 24 h, the slides were rinsed with water and stained with SYTO9 (Molecular Probes) for 20 min in the dark. Slides were then rinsed with water to remove excess stain. Biofilm bacteria were visualized at 10× magnification using a Nikon Eclipse 80i fluorescent microscope.

### Statistics and Reproducibility of Results

All microtiter plate assays were performed in duplicate wells, which exhibited an average variation of <10%. All assays were performed 2–3 times with similarly significant differences in absorbance values. The significance of differences between means was measured using the Student’s *t*-test. A *P* value of ≤0.05 was considered significant.

## Results

### *A. pleuropneumoniae* Colony Biofilm Extract Exhibits Antibiofilm Activity

**Figure 1:**
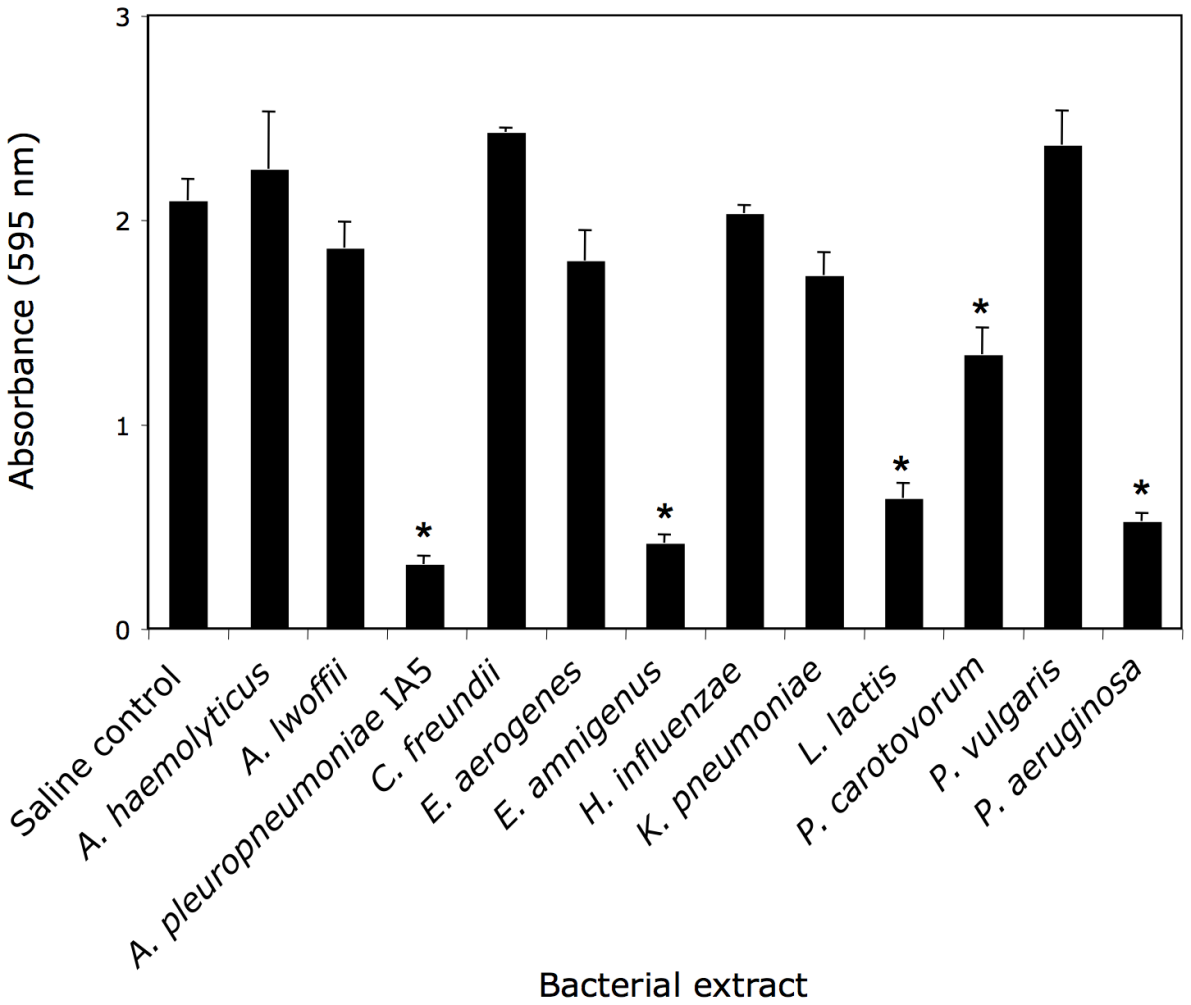
*S. aureus* biofilm formation in the presence of 12 different cell-free colony biofilm extracts. *S. aureus* was cultured in the presence 10% extract isolated from the bacteria indicated along the bottom. Control cultures were incubated with 10% saline. After 18 h, biofilms were rinsed and stained with crystal violet. Values show the average amount of crystal violet binding for duplicate wells and error bars indicate range. Asterisks indicate absorbance values significantly different from saline control (*P*<0.05).

**Figure 2:**
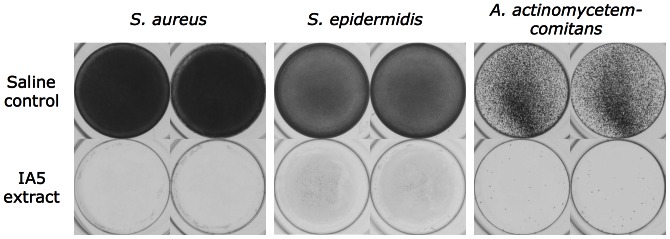
Biofilm formation by *S. aureus*, *S. epidermidis* and *A. actinomycetemcomitans* in the presence of *A. pleuropneumoniae* colony biofilm extract. Inocula were supplemented with 10% *A. pleuropneumoniae* IA5 extract, or 10% saline as a control. Bacteria were cultured in 96-well microtiter plates. After 18 h, biofilms were rinsed, stained with crystal violet, and photographed. Duplicate wells are shown.

We isolated colony biofilm extracts from 12 different bacteria and tested the extracts for their ability to inhibit biofilm formation by *S. aureus* in a 96-well microtiter plate assay (Fig. 1). The bacteria that were tested comprised a convenience sample of 11 *Proteobacteria* and *Lactococcus lactis* (Table 1). Extracts were tested at a concentration of 10% by vol. Under these conditions, five extracts significantly inhibited *S. aureus* biofilm formation, while seven extracts had no significant effect on biofilm formation. Inhibition of *S. aureus* biofilm formation by *P. aeruginosa* extract was partially due to growth inhibition (data not shown). We selected *A. pleuropneumoniae* IA5 colony biofilm extract for further analysis because it exhibited a high level of biofilm inhibition, but it did not inhibit *S. aureus* growth or contain proteases or DNases that are known to inhibit *S. aureus* biofilm formation (data not shown). *A. pleuropneumoniae* IA5 colony biofilm extract inhibited biofilm formation by *S. aureus*, *S. epidermidis*, and the Gram-negative *Aggregatibacter actinomycetemcomitans* (Fig. 2). *A. pleuropneumoniae* colony biofilm extract did not inhibit the growth of *S. aureus*, *S. epidermidis* or *A. actinomycetemcomitans* (data not shown).

### The Antibiofilm Activity in *A. pleuropneumoniae* Colony Biofilm Extract is due to Capsular Polysaccharide

**Figure 3:**
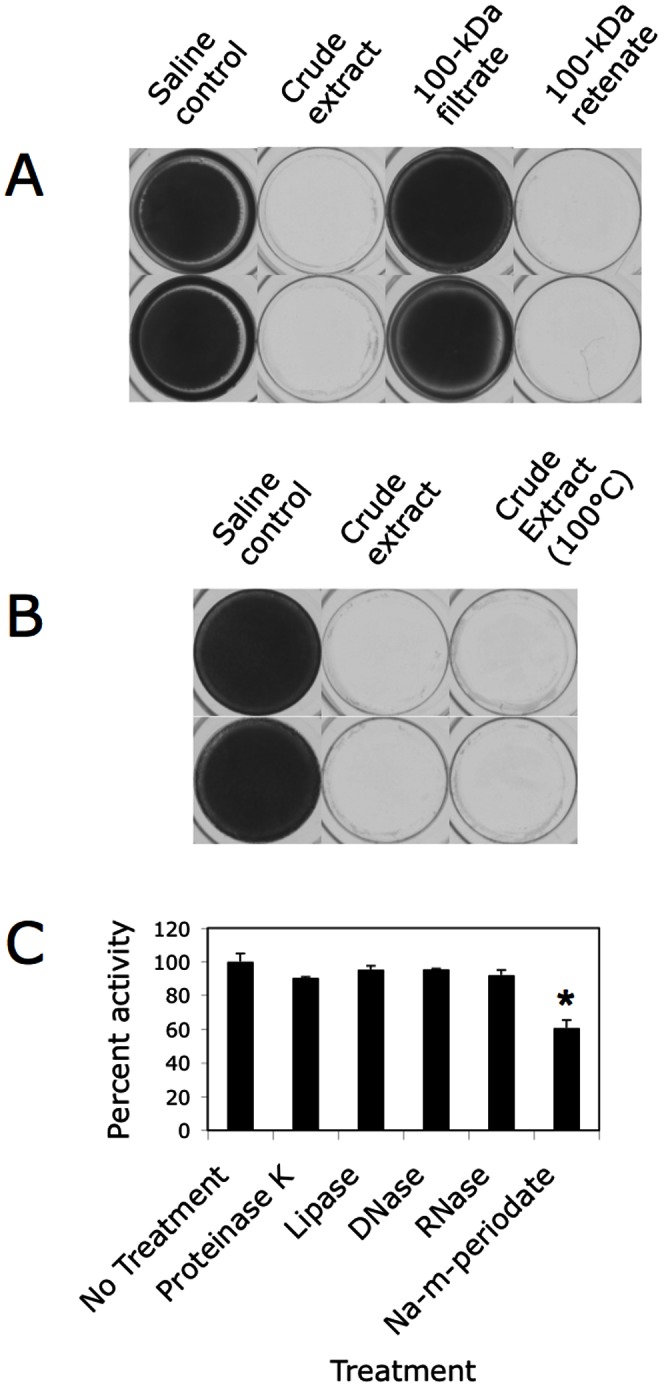
Physical and chemical analyses of the antibiofilm activity in *A. pleuropneumoniae* IA5 colony biofilm extract. (**A**) Biofilm formation by *S. aureus* in the presence of 10% saline (control), crude extract, or the filtrate and retenate of crude extract passed through a 100-kDa pore-size filter. Duplicate wells are shown. (**B**) Biofilm formation by *S. aureus* in the presence of 10% saline, crude extract, or crude extract that was incubated at 100°C for 15 min. Duplicate wells are shown. (**C**) Biofilm-inhibiting activity of IA5 extract treated with proteinase K, lipase, DNase, RNase or sodium metaperiodate. Biofilm inhibition was measured against *S. aureus* as in panels A and B. Percent activity was calculated as the ratio of the reduction in crystal violet absorbance exhibited by the treated extract to the reduction in absorbance exhibited by the mock-treated extract (Δ*A*_595(treated extract)_/Δ*A*_595(mock-treated extract)_ × 100). The graph show mean and range percent activity values from 2-3 experiments. Asterisk indicates a significant reduction in activity (*P*<0.05).

Physical analysis of the *A. pleuropneumoniae* IA5 colony biofilm extract indicated that the antibiofilm activity in the extract was >100 kDa in mass (Fig. 3A) and heat stable (Fig. 3B). Treatment of the extract with proteinase K, lipase, DNase or RNase had no effect on its antibiofilm activity (Fig. 3C). In contrast, treatment of the extract with the carbohydrate-active agent sodium metaperiodate significantly reduced its antibiofilm activity (Fig. 3C). These data suggest that the antibiofilm activity in the *A. pleuropneumoniae* colony biofilm extract was due to high-molecular-weight polysaccharide.

**Figure 4:**
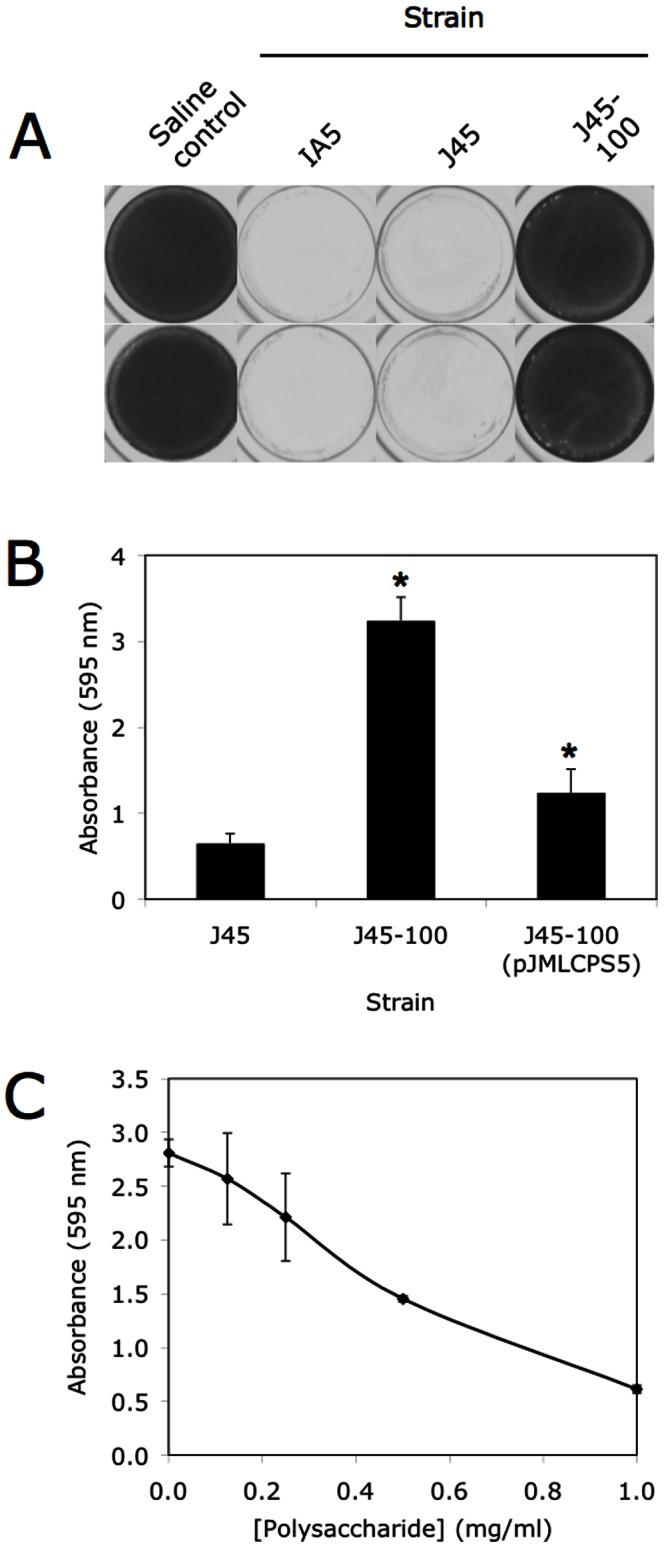
Biofilm formation by *S. aureus* in the presence of *A. pleuropneumoniae* serotype 5 wild-type and capsule-mutant colony biofilm extracts and purified serotype 5 capsular polysaccharide. (**A**) *S. aureus* biofilm formation in the presence of colony biofilm extracts isolated from wild-type strains IA5 and J45, and isogenic J45 capsule-mutant J45-100. Duplicate wells are shown. *, significantly different from J45 control extract (*P*<0.05). (**B**) Quantitation of *S. aureus* biofilm formation in the presence of extracts isolated from wild-type J45, capsule mutant J45-100, and genetically-complemented J45-100 capsule-mutant. (**C**) Quantitation of *S. aureus* biofilm formation in the presence of purified serotype 5 capsular polysaccharide. Values in panels B and C show averages for duplicate wells and error bars indicate range.

To determine whether the *A. pleuropneumoniae* serotype 5 capsule contributes to the antibiofilm activity of the colony biofilm extract, we isolated extracts from wild-type serotype 5 strain J45, and from the isogenic serotype 5 capsule-mutant strain J45-100. Like extracts isolated from strain IA5, extracts isolated from strain J45 inhibited *S. aureus* biofilm formation (Fig. 4A). In contrast, extracts isolated from capsule-mutant strain J45-100 did not inhibit *S. aureus* biofilm formation (Fig. 4A). Extracts isolated from capsule-mutant strain J45-100 transformed with plasmid pJMLCPS5, which restores capsule production, exhibited significantly greater antibiofilm activity than extracts isolated from uncomplemented J45-100 (Fig. 4B). This biofilm inhibition was not due to growth inhibition caused by antibiotic carryover from the genetically-complemented *A. pleuropneumoniae* culture (data not shown). In addition, purified serotype 5 capsular polysaccharide inhibited *S. aureus* biofilm formation in a dose-dependent manner (Fig. 4C). These findings confirm that the *A. pleuropneumoniae* serotype 5 capsule exhibits antibiofilm activity against *S. aureus*.

### *A. pleuropneumoniae* Colony Biofilm Extract Inhibits *S. aureus* Cell-to-cell and Cell-to-Surface Interactions

**Figure 5:**
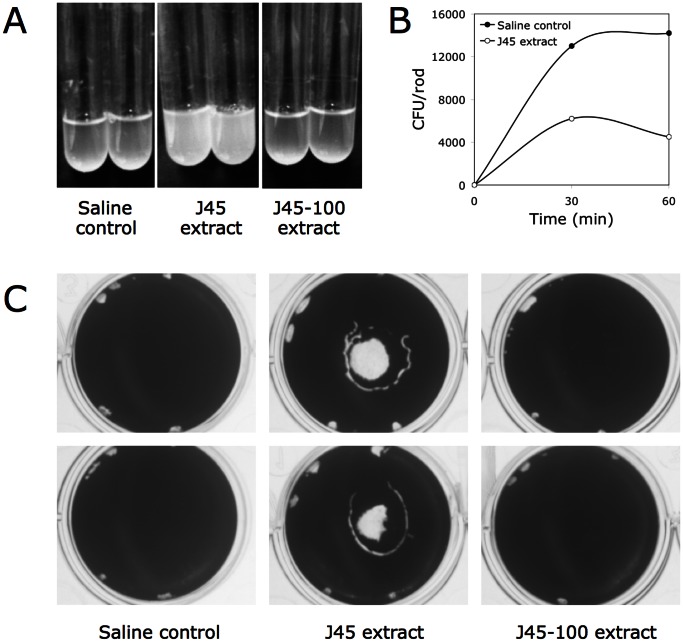
*A. pleuropneumoniae* serotype 5 colony biofilm extract exhibits surfactant-like properties. (**A**) Intercellular adhesion of *S. aureus* planktonic cells cultured in 10% saline (control), wild-type J45 extract, or capsule-mutant J45-100 extract. Duplicate tubes are shown. (**B**) Attachment of *S. aureus* planktonic cells to stainless steel rods in the presence of 10% *A. pleuropneumoniae* J45 colony biofilm extract. Values show average for duplicate rods. (**C**) Biofilm formation by *S. aureus* in polystyrene microtiter plate wells coated with saline, J45 extract, or J45-100 extract. Duplicate wells are shown.

*S. aureus* cells cultured in broth with shaking aggregated and settled to the bottom of the tube, resulting in a visible clearing of the broth (Fig. 5A, left panel). When *A. pleuropneumoniae* J45 extract was present in the culture, the *S. aureus* cells exhibited less settling (Fig. 5A, middle panel). *S. aureus* cells cultured in the presence of extract isolated from serotype 5 capsule-mutant strain J45-100 exhibited the same amount of settling as control cultures (Fig. 5A, right panel). These data suggest that *A. pleuropneumoniae* serotype 5 capsule inhibits *S. aureus* intercellular adhesion.

We also measured binding of *S. aureus* planktonic cells to stainless steel rods in the presence and absence of *A. pleuropneumoniae* J45 colony biofilm extract. As shown in Fig. 5B, the *A. pleuropneumoniae* extract significantly inhibited *S. aureus* cell binding after both 30 and 60 min.

We also tested whether *A. pleuropneumoniae* J45 colony biofilm extract could modify the surface properties of an abiotic substrate. To do this, we used evaporation coating to deposit the extract onto the surface of polystyrene wells, and then tested the ability of the coated surfaces to resist biofilm formation by *S. aureus*. When *A. pleuropneumoniae* extract was applied to the polystyrene surfaces, the coated surfaces efficiently repelled *S. aureus* biofilm formation in the area where the extract was deposited (Fig. 5C). Surfaces coated with extract isolated from the serotype 5 capsule-mutant strain did not resist *S. aureus* biofilm formation (Fig. 5C).

### *A. pleuropneumoniae* Colony Biofilm Extract does not Inhibit *A. pleuropneumoniae* Biofilm Formation

**Figure 6:**
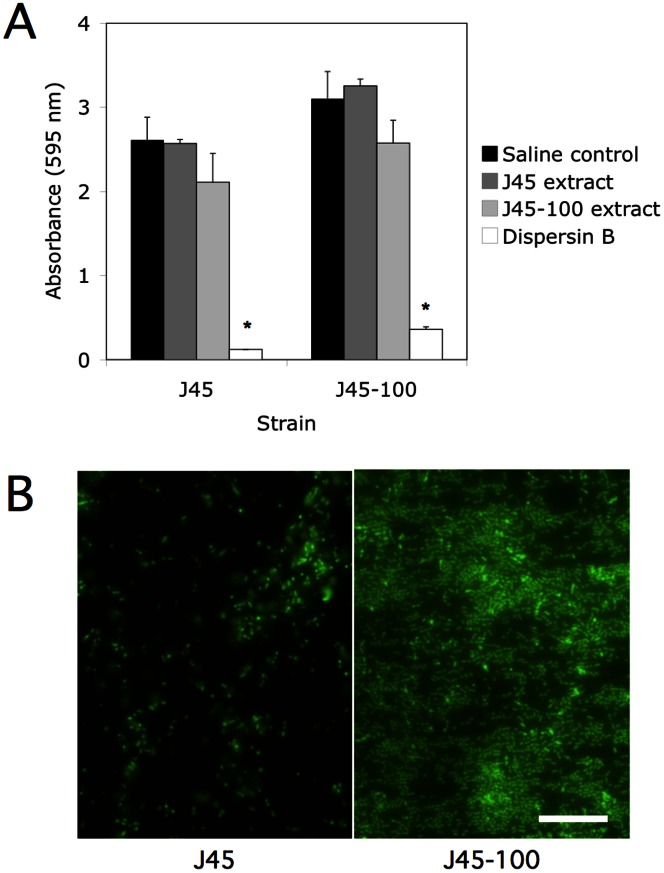
Biofilm formation by ***A. pleuropneumoniae*** wild-type J45 and capsule-mutant J45-100. (**A**) Quantitation of biofilm formation in 96-well polystyrene microtiter plates. Biofilms were grown in the presence of 10% saline (control), J45 colony biofilm extract, J45-100 colony biofilm extract, or 10 mg/L dispersin B. Biofilms were stained with crystal violet. Values show mean absorbance values and standard deviation from four independent experiment. *, significantly different from saline control (*P*<0.05). (**B**) Micrographs of 24-h-old biofilms cultured on glass slides. Cells were stained with SYTO9. Measure bar = 20 µm.

Extracts isolated from *A. pleuropneumoniae* wild-type strain J45 and capsule-mutant strain J45-100 were tested for their ability to inhibit biofilm formation by *A. pleuropneumoniae* J45 and J45-100. There was no significant difference between the amount of biofilm formed by strains J45 and J45-100 when quantitated by spectrophotometry (Fig. 6A). Neither the J45 nor the J45-100 extract significantly inhibited biofilm formation by either strain. Dispersin B significantly inhibited biofilm formation by both strains, indicating that strain J45, like *A. pleuropneumoniae* serotype 5 strain IA5 [23], produces PNAG-dependent biofilms. Microscopic analysis revealed that biofilms produced by the capsule mutant strain J45-100 appeared much denser than biofilms produced by wild-type strain J45 (Fig. 6B).

## Discussion

The Gram-negative bacterium *A. pleuropneumoniae* is the causative agent of swine pleuropneumonia, a severe and contagious respiratory disease that affects pigs worldwide [24]. *A. pleuropneumoniae* strains are divided into 15 serotypes based on the structures of their capsular polysaccharide (CPS) [25]. Serotype 5 is a prevalent *A. pleuropneumoniae* serotype in the U.S., Canada, Brazil, Chile, Korea and Taiwan [26]. *A. pleuropneumoniae* serotype 5 CPS consists of a linear polymer with the structure →6)-α-D-Glc*p*NAc-(1→5)-β-D-dOclA*p*-(2→ [27]. A subset of serotype 5 strains (designated serotype 5b) contain an additional β-D-Glc*p* reside covalently joined to the β-D-dOclA*p* residue in (1→4) linkage [26]. Mutant strains lacking serotype 5 CPS have been shown to exhibit decreased serum resistance *in vitro* and decreased virulence in pigs [28], [29].

Our findings demonstrate that *A. pleuropneumoniae* serotype 5 CPS exhibits nonbiocidal antibiofilm activity against other Gram-negative and Gram-positive bacteria. Several other bacteria produce nonbiocidal antibiofilm polysaccharides including *Kingella kingae* [11], *Escherichia coli* [8], [19], *Bacillus licheniformis* [30], *Lactobacillus acidophilus* [31], *Streptococcus phocae* [32] and *Vibrio* sp. [33]. The biological functions of these antibiofilm polysaccharides are not known, but they may include water channel formation or biofilm dispersal [34]. The fact that the *A. pleuropneumoniae* CPS mutant strain exhibited increased biofilm formation compared to the wild-type strain (Fig. 6B) suggests that the serotype 5 capsule may function in water channel formation or biofilm dispersal. Previous studies showed that *A. pleuropneumoniae* produces dispersin B, which may also function in maintaining biofilm architecture or in biofilm dispersal [23].

We found that *A. pleuropneumoniae* serotype 5 CPS inhibited biofilm formation by *S. aureus*, *S. epidermidis* and *A. actinomycetemcomitans*, but not by *A. pleuropneumoniae* serotype 5 itself. Biofilm formation by all four of these species is dependent on the production of poly-*N*-acetylglucosamine (PNAG) matrix polysaccharide [20], [35]. Although PNAG is the major biofilm matrix adhesin in both *A. pleuropneumoniae* and *S. epidermidis* biofilms, *A. pleuropneumoniae* serotype 5 CPS inhibited only *S. epidermidis* biofilm formation and not *A. pleuropneumoniae* serotype 5 biofilm formation. In addition, colony biofilm extracts isolated from a PNAG-deficient strain of *A. pleuropneumoniae* serotype 5 exhibited the same antibiofilm activity as extracts isolated from a wild-type strain (unpublished results). These observations suggest that the antibiofilm activity of serotype 5 CPS is independent of the intercellular adhesion activity of PNAG.

Our screen of colony biofilm extracts isolated from 12 phylogenetically diverse bacteria identified five extracts that exhibited antibiofilm activity against *S. aureus*. In a similar screen, Rendueles *et al*. [19] found that 20% of cell-free biofilm extracts isolated from 122 natural *E. coli* isolates exhibited antibiofilm activity against a panel of seven biofilm-forming Gram-positive and Gram-negative bacteria. These findings suggest that bacterial biofilms constitute untapped sources of natural bioactive molecules antagonizing adhesion or biofilm formation of other bacteria.

## References

[1] Hall-Stoodley L, Costerton JW, Stoodley P (2004) Bacterial biofilms: from the natural environment to infectious diseases. Nat Rev Microbiol 2: 95–108. 15040259 10.1038/nrmicro821

[2] Parsek MR, Singh PK (2003) Bacterial biofilms: an emerging link to disease pathogenesis. Annu Rev Microbiol 57: 677–701. 14527295 10.1146/annurev.micro.57.030502.090720

[3] Rice SA, Koh KS, Queck SY, Labbate M, Lam KW, et al (2005) Biofilm formation and sloughing in *Serratia marcescens* are controlled by quorum sensing and nutrient cues. J Bacteriol 187: 3477–3485. 15866935 10.1128/JB.187.10.3477-3485.2005 PMC1111991

[4] Schooling SR, Charaf UK, Allison DG, Gilbert P (2004) A role for rhamnolipid in biofilm dispersion. Biofilms 1: 91–99.

[5] Davey ME, Caiazza NC, O’Toole GA (2003) Rhamnolipid surfactant production affects biofilm architecture in *Pseudomonas aeruginosa* PAO1. J Bacteriol 185: 1027–1036. 12533479 10.1128/JB.185.3.1027-1036.2003 PMC142794

[6] Kaplan JB, Ragunath C, Ramasubbu N, Fine DH (2003) Detachment of *Actinobacillus actinomycetemcomitans* biofilm cells by an endogenous beta-hexosaminidase activity. J Bacteriol 185: 4693–4698. 12896987 10.1128/JB.185.16.4693-4698.2003 PMC166467

[7] Mann EE, Rice KC, Boles BR, Endres JL, Ranjit D, et al. (2009) Modulation of eDNA release and degradation affects *Staphylococcus aureus* biofilm maturation. PLoS One 4, e5822. 10.1371/journal.pone.0005822 PMC2688759 19513119

[8] Valle J, Da Re S, Henry N, Fontaine T, Balestrino D, et al.. (2006) Broad-spectrum biofilm inhibition by a secreted bacterial polysaccharide. Proc Natl Acad Sci U S A 103, 12558–12563. 10.1073/pnas.0605399103 PMC1567917 16894146

[9] Kolodkin-Gal I, Romero D, Cao S, Clardy J, Kolter R, et al.. (2010) D-amino acids trigger biofilm disassembly. Science 328, 627–629. 10.1126/science.1188628 PMC2921573 20431016

[10] Kaplan JB (2010) Biofilm dispersal: mechanisms, clinical implications, and potential therapeutic uses. J Dent Res 89, 205–218. 10.1177/0022034509359403 PMC3318030 20139339

[11] Bendaoud M, Vinogradov E, Balashova NV, Kadouri DE, Kachlany SC, et al. (2011) Broad-spectrum biofilm inhibition by *Kingella kingae* exopolysaccharide. J Bacteriol 193, 3879–3886. 10.1128/JB.00311-11 PMC3147541 21602333

[12] Merritt JH, Kadouri DE, O’Toole GA (2005) Growing and analyzing static biofilms. Curr Protoc Microbiol Chapter 1, Unit 1B 1. 10.1002/9780471729259.mc01b01s00 PMC4568995 18770545

[13] Auerbach ID, Sorensen C, Hansma HG, Holden PA (2000) Physical morphology and surface properties of unsaturated *Pseudomonas putida* biofilms. J Bacteriol 182, 3809–3815. 10.1128/jb.182.13.3809-3815.2000 PMC94554 10850998

[14] Dietrich LE, Teal TK, Price-Whelan A, Newman DK (2008) Redox-active antibiotics control gene expression and community behavior in divergent bacteria. Science 321, 1203–1206. 10.1126/science.1160619 PMC2745639 18755976

[15] Kim J, Park HJ, Lee JH, Hahn JS, Gu MB, et al.. (2009) Differential effect of chlorine on the oxidative stress generation in dormant and active cells within colony biofilm. Water Res 43, 5252–5259. 10.1016/j.watres.2009.08.044 19781733

[16] Mah TF, Pitts B, Pellock B, Walker GC, Stewart PS, et al. (2003) A genetic basis for *Pseudomonas aeruginosa* biofilm antibiotic resistance. Nature 426, 306–310. 10.1038/nature02122 14628055

[17] Ramsey MM, Whiteley M (2009) Polymicrobial interactions stimulate resistance to host innate immunity through metabolite perception. Proc Natl Acad Sci U S A 106, 1578–1583. 10.1073/pnas.0809533106 PMC2629492 19164580

[18] Rani SA, Pitts B, Beyenal H, Veluchamy RA, Lewandowski Z, et al.. (2007) Spatial patterns of DNA replication, protein synthesis, and oxygen concentration within bacterial biofilms reveal diverse physiological states. J Bacteriol 189, 4223–4233. 10.1128/JB.00107-07 PMC1913414 17337582

[19] Rendueles O, Travier L, Latour-Lambert P, Fontaine T, Magnus J, et al. (2011) Screening of *Escherichia coli* species biodiversity reveals new biofilm-associated antiadhesion polysaccharides. MBio 2, e00043–00011. 10.1128/mBio.00043-11 PMC3101779 21558434

[20] Izano EA, Amarante MA, Kher WB, Kaplan JB (2008) Differential roles of poly-N-acetylglucosamine surface polysaccharide and extracellular DNA in *Staphylococcus aureus* and *Staphylococcus epidermidis* biofilms. Appl Environ Microbiol 74, 470–476. 10.1128/AEM.02073-07 PMC2223269 18039822

[21] Inzana TJ (1987) Purification and partial characterization of the capsular polymer of *Haemophilus pleuropneumoniae* serotype 5. Infect Immun 55, 1573–1579. 10.1128/iai.55.7.1573-1579.1987 PMC260560 3596801

[22] Kaplan JB, Fine DH (2002) Biofilm dispersal of *Neisseria subflava* and other phylogenetically diverse oral bacteria. Appl Environ Microbiol 68, 4943–4950. 10.1128/AEM.68.10.4943-4950.2002 PMC126399 12324342

[23] Kaplan JB, Velliyagounder K, Ragunath C, Rohde H, Mack D, et al. (2004) Genes involved in the synthesis and degradation of matrix polysaccharide in *Actinobacillus actinomycetemcomitans* and *Actinobacillus pleuropneumoniae* biofilms. J Bacteriol 186, 8213–8220. 10.1128/JB.186.24.8213-8220.2004 PMC532409 15576769

[24] Bossé JT, Janson H, Sheehan BJ, Beddek AJ, Rycroft AN, et al. (2002) *Actinobacillus pleuropneumoniae*: pathobiology and pathogenesis of infection. Microbes Infect 4, 225–235. 10.1016/s1286-4579(01)01534-9 11880056

[25] Jacques M (2004) Surface polysaccharides and iron-uptake systems of *Actinobacillus pleuropneumoniae*. Can J Vet Res 68, 81–85. PMC1142149 15188950

[26] Dubreuil JD, Jacques M, Mittal KR, Gottschalk M (2000) *Actinobacillus pleuropneumoniae* surface polysaccharides: their role in diagnosis and immunogenicity. Anim Health Res Rev 1, 73–93. 10.1017/s1466252300000074 11708600

[27] Altman E, Brisson JR, Perry MB (1987) Structure of the capsular polysaccharide of *Haemophilus pleuropneumoniae* serotype 5. Eur J Biochem 170, 185–192. 10.1111/j.1432-1033.1987.tb13685.x 3691518

[28] Inzana TJ, Todd J, Veit HP (1993) Safety, stability, and efficacy of noncapsulated mutants of *Actinobacillus pleuropneumoniae* for use in live vaccines. Infect Immun 61, 1682–1686. 10.1128/iai.61.5.1682-1686.1993 PMC280751 8478056

[29] Ward CK, Lawrence ML, Veit HP, Inzana TJ (1998) Cloning and mutagenesis of a serotype-specific DNA region involved in encapsulation and virulence of *Actinobacillus pleuropneumoniae* serotype 5a: concomitant expression of serotype 5a and 1 capsular polysaccharides in recombinant *A. pleuropneumoniae* serotype 1. Infect Immun 66, 3326–3336. 10.1128/iai.66.7.3326-3336.1998 PMC108349 9632602

[30] Sayem SM, Manzo E, Ciavatta L, Tramice A, Cordone A, et al. (2011) Anti-biofilm activity of an exopolysaccharide from a sponge-associated strain of *Bacillus licheniformis*. Microb Cell Fact 10, 74. 10.1186/1475-2859-10-74 PMC3196911 21951859

[31] Kim Y, Oh S, Kim SH (2009) Released exopolysaccharide (r-EPS) produced from probiotic bacteria reduce biofilm formation of enterohemorrhagic *Escherichia coli* O157:H7. Biochem Biophys Res Commun 379, 324–329. 10.1016/j.bbrc.2008.12.053 19103165

[32] Kanmani P, Satish kumar R, Yuvaraj N, Paari KA, Pattukumar V, et al. (2011) Production and purification of a novel exopolysaccharide from lactic acid bacterium *Streptococcus phocae* PI80 and its functional characteristics activity in vitro. Bioresour Technol 102, 4827–4833. 10.1016/j.biortech.2010.12.118 21300540

[33] Jiang P, Li J, Han F, Duan G, Lu X, et al (2011) Antibiofilm activity of an exopolysaccharide from marine bacterium *Vibrio* sp QY101. PLoS One 6: e18514. 21490923 10.1371/journal.pone.0018514 PMC3072402

[34] Rendueles R, Kaplan JB, Ghigo JM (2013) Antibiofilm polysaccharides. Environ Microbiol 15, 334–346. 10.1111/j.1462-2920.2012.02810.x PMC3502681 22730907

[35] Kaplan JB, Ragunath C, Velliyagounder K, Fine DH, Ramasubbu N (2004) Enzymatic detachment of *Staphylococcus epidermidis* biofilms. Antimicrob Agents Chemother 48, 2633–2636. 10.1128/AAC.48.7.2633-2636.2004 PMC434209 15215120

[36] Fine DH, Furgang D, Schreiner HC, Goncharoff P, Charlesworth J, et al. (1999) Phenotypic variation in *Actinobacillus actinomycetemcomitans* during laboratory growth: implications for virulence. Microbiology 145, 1335–1347. 10.1099/13500872-145-6-1335 10411260

[37] Izano EA, Shah SM, Kaplan JB (2009) Intercellular adhesion and biocide resistance in nontypeable *Haemophilus influenzae* biofilms. Microb Pathog 46, 207–213. 10.1016/j.micpath.2009.01.004 PMC2691864 19490830

[38] Horsburgh M, Aish J, White I, Shaw L, Lithgow J, et al. (2002) σ^B^ modulates virulence determinant expression and stress resistance: characterization of a functional *rsbU* strain derived from *Staphylococcus aureus* 8325–4. J Bacteriol 184, 5457–5467. 10.1128/JB.184.19.5457-5467.2002 PMC135357 12218034

